# *Vital Signs*: Prevalence of Key Cardiovascular Disease Risk Factors for Million Hearts 2022 — United States, 2011–2016

**DOI:** 10.15585/mmwr.mm6735a4

**Published:** 2018-09-07

**Authors:** Hilary K. Wall, Matthew D. Ritchey, Cathleen Gillespie, John D. Omura, Ahmed Jamal, Mary G. George

**Affiliations:** ^1^Division for Heart Disease and Stroke Prevention, CDC; ^2^Division of Nutrition, Physical Activity, and Obesity, CDC; ^3^Office on Smoking and Health, CDC.

## Abstract

**Introduction:**

Despite decades-long reductions in cardiovascular disease (CVD) mortality, CVD mortality rates have recently plateaued and even increased in some subgroups, and the prevalence of CVD risk factors remains high. Million Hearts 2022, a 5-year initiative, was launched in 2017 to address this burden. This report establishes a baseline for the CVD risk factors targeted for reduction by the initiative during 2017–2021 and highlights recent changes over time.

**Methods:**

Risk factor prevalence among U.S. adults was assessed using data from the National Health and Nutrition Examination Survey, National Survey on Drug Use and Health, and National Health Interview Survey. Multivariate analyses were performed to assess differences in prevalence during 2011–2012 and the most recent cycle of available data, and across subgroups.

**Results:**

During 2013–2014, the prevalences of aspirin use for primary and secondary CVD prevention were 27.4% and 74.9%, respectively, and of statin use for cholesterol management was 54.5%. During 2015–2016, the average daily sodium intake was 3,535 mg/day and the prevalences of blood pressure control, combustible tobacco use, and physical inactivity were 48.5%, 22.3%, and 29.1%, respectively. Compared with 2011–2012, significant decreases occurred in the prevalences of combustible tobacco use and physical inactivity; however, a decrease also occurred for aspirin use for primary or secondary prevention. Disparities in risk factor prevalences were observed across age groups, genders, and racial/ethnic groups.

**Conclusions and Implications for Public Health Practice:**

Millions of Americans have CVD risk factors that place them at increased risk for having a cardiovascular event, despite the existence of proven strategies for preventing or managing CVD risk factors. A concerted effort to implement these strategies will be needed to prevent one million acute cardiovascular events during the 5-year initiative.

## Introduction

Despite steady declines in CVD mortality rates over approximately the last 40 years, heart disease and stroke remain the first and fifth leading causes of death in the United States, respectively, and their associated mortality rates have recently begun to plateau in the general population and even increase among some subpopulations. (*1**–**3*) Furthermore, CVD annually accounts for approximately $330 billion in direct and indirect costs in the United States: approximately one in seven health care dollars is spent on CVD (*4*). To address this burden, in 2012, the U.S. Department of Health and Human Services launched Million Hearts, a national initiative co-led by CDC and the Centers for Medicare & Medicaid Services, with the goal of preventing one million acute cardiovascular events over 5 years. Because important groundwork and progress were made during the first 5 years (*5**,**6*), Million Hearts 2022 was launched in 2017 to accelerate the implementation of effective strategies to improve cardiovascular health.

During 2017–2021, Million Hearts 2022 priorities are keeping adults healthy through community-based strategies that reduce combustible tobacco use, sodium intake, and physical inactivity as well as optimizing health care for those with and at risk for CVD through clinical strategies that improve appropriate aspirin use, blood pressure control, cholesterol management, tobacco cessation, and participation in cardiac rehabilitation.* Million Hearts 2022 also has a special focus on selected priority populations at risk, including blacks/African Americans with hypertension, adults aged 35–64 years for whom heart disease mortality rates are rising, adults who have had a previous heart attack or stroke, and persons with mental health or substance use disorders who use tobacco (*7*). This report uses several national surveillance systems to provide baseline data and describe recent changes for key CVD risk factors for which accelerated progress must be made to achieve national goals.

## Methods

Data for this report were gathered from three national surveillance systems: the National Health and Nutrition Examination Survey (NHANES^†^), the National Survey on Drug Use and Health (NSDUH^§^), and the National Health Interview Survey (NHIS^¶^). The details for all three surveys have been published previously (*8**–**10*).

NHANES is a complex survey of a multistage probability sample of the civilian, noninstitutionalized U.S. population that combines interviews and physical examinations. Data from NHANES from 2011 to 2014 were used to calculate prevalence estimates for aspirin use for primary CVD prevention** among adults aged 50–59 years, aspirin use for secondary CVD prevention^††^ among adults aged ≥40 years, combined aspirin use “as appropriate”^§§^ among adults aged ≥40 years, and statin use among eligible adults aged ≥21 years.^¶¶^ Mean daily sodium intake (mg/day)*** among adults aged ≥18 years and blood pressure control^†††^ estimates among adults aged ≥18 years with hypertension were calculated using 2011–2016 NHANES data.

NSDUH is an annual nationwide survey that collects information through face-to-face household interviews about the use of illicit drugs, alcohol, and tobacco among the noninstitutionalized U.S. population aged ≥12 years. Data from the 2011–2016 NSDUH were combined into 2-year cycles to estimate the prevalence of current combustible tobacco use^§§§^ among adults aged ≥18 years.

NHIS is an annual, nationally representative, in-person survey of the noninstitutionalized U.S. civilian population. Data from the 2011–2016 NHIS were combined into 2-year cycles to estimate the prevalence of physical inactivity^¶¶¶^ among adults aged ≥18 years.

Up to three survey cycles (2011–2012, 2013–2014, and 2015–2016) were examined using sex-, age-, and race/ethnicity-adjusted regression analyses. Sex-, age-, and race/ethnicity-adjusted t-tests were used to examine prevalence changes comparing 2011–2012 with the most recent data cycle and differences between sex, age, and racial/ethnic groups within the most recent data cycle. Results were considered significant for p-values <0.05.

## Results

**FIGURE 1:**
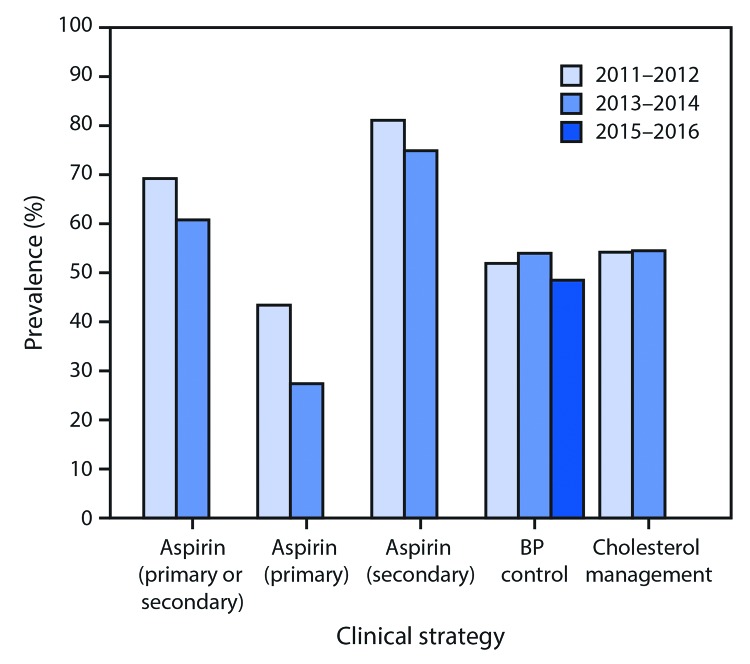
Prevalence of Million Hearts 2022 clinical strategies*^,^^†^^,^^§^ to prevent cardiovascular disease among adults^¶^^,^** — United States, 2011–2012, 2013–2014, and 2015–2016 **Source:** National Health and Nutrition Examination Survey, National Center for Health Statistics, CDC. **Abbreviation:** BP = blood pressure. * Aspirin use was defined by an answer of “yes” to the question “Doctors and other health care providers sometimes recommend that you take a low-dose aspirin each day to prevent heart attacks, strokes, or cancer. Have you ever been told to do this?” and an answer of “yes” or “sometimes” to the question “Are you/ now following this advice?”; an answer of “yes” to the question “On your own, are you now taking a low-dose aspirin each day to prevent heart attacks, strokes, or cancer?”; or aspirin identified in the prescription medication data files. Participants who reported taking an anticoagulant in the prescription medication files but not taking aspirin were excluded. Aspirin use for primary prevention includes examined adults aged 50–59 years without a history of cardiovascular disease (CVD) and with an American College of Cardiology/American Heart Association 10-year atherosclerotic CVD risk score ≥10%. Aspirin use of secondary prevention includes examined adults aged ≥40 years with a history of CVD. ^†^ BP control was defined as an average systolic BP <140 mm Hg and an average diastolic BP <90 mm Hg among adults aged ≥18 years with hypertension. Hypertension is defined as an average systolic BP ≥140 mm Hg, or an average diastolic BP ≥90 mm Hg, or self-reported current use of BP-lowering medication. ^§^ Cholesterol management is defined as current statin use, based on the prescription medication data files, among fasting adults aged ≥21 years for whom statin therapy is recommended. ^¶^ For aspirin (primary or secondary), t-test p-value <0.01 comparing 2013–2014 with 2011–2012, adjusted for sex, age group, and race/ethnicity. ** For aspirin (primary), t-test p-value <0.05 comparing 2013–2014 with 2011–2012, adjusted for sex and race/ethnicity.

**Clinical Strategies**. During 2013–2014, the prevalences of recommended aspirin use for primary and secondary CVD prevention were 27.4% and 74.9%, respectively, with a significant decrease from 2011–2012 for primary prevention (43.4%) but not for secondary prevention (Table 1) (Figure 1). Combined, the prevalence of aspirin use “when appropriate” was 60.8%, which represents a significant decline from 69.2%, during 2011–2012 (Figure 1) and equates to an estimated 9.0 million persons not taking aspirin as recommended. The prevalence of aspirin use for secondary prevention was higher among adults aged ≥65 years (81.4%) than among those aged 40–64 years (63.2%), and among non-Hispanic whites (whites) (77.9%) compared with Hispanics (51.5%). The overall prevalence of recommended aspirin use when appropriate was higher among adults aged ≥65 years than among those aged 40–64 years.

**TABLE 1:** Current prevalence of Million Hearts 2022 clinical strategies to prevent cardiovascular disease among adults — United States, 2013–2014 and 2015–2016

Clinical strategy/Demographic group	% (SE)	(95% CI)	No. (millions)*	t-test p-value^†^
**Aspirin use^§^ when appropriate for primary or secondary prevention^¶^ among adults aged ≥40 years — NHANES, 2013–2014**				
**Total**	**60.8 (2.1)**	**(56.5–64.9)**	**14.0**	**—**
**Sex**				
Male	58.0 (2.8)	(52.2–63.5)	8.5	reference
Female	65.6 (3.3)	(58.6–72.0)	5.4	0.566
**Age group (yrs)**				
40–64	43.7 (3.3)	(37.1–50.4)	5.4	reference
65–74	78.9 (4.3)	(68.9–86.3)	4.6	<0.001
≥65	81.4 (2.7)	(75.3–86.2)	8.8	<0.001
≥75	84.8 (3.1)	(77.4–90.1)	4.3	<0.001
**Race/Ethnicity**				
White, non-Hispanic	65.9 (2.1)	(61.5–70.1)	10.7	reference
Black, non-Hispanic	51.0 (5.3)	(40.5–61.5)	1.8	0.621
Asian, non-Hispanic	42.2 (8.8)	(26.0–60.2)	0.4	0.016
Hispanic	45.4 (3.6)	(38.3–52.6)	0.9	0.061
Other	56.2 (15.7)	(26.1–82.3)	0.2	0.348
**Aspirin use^§^ when appropriate for primary prevention^¶^ among adults aged 50–59 years — NHANES, 2013–2014**				
**Total**	**27.4 (4.1)**	**(20.0–36.3)**	**1.9**	**—**
**Sex**				
Male	27.6 (4.4)	(19.7–37.1)	1.6	reference
Female	26.6 (6.0)	(16.3–40.2)	0.3	0.688
**Race/Ethnicity**				
White, non-Hispanic	27.9 (4.1)	(20.3–36.9)	1.1	reference
Black, non-Hispanic	28.8 (6.8)	(17.2–44.0)	0.6	0.809
Asian, non-Hispanic	—**	—**	—**	—**
Hispanic	32.4 (9.7)	(16.4–54.0)	0.2	0.617
Other	—**	—**	—**	—**
**Aspirin use^§^ when appropriate for secondary prevention^¶^ among adults aged ≥40 years — NHANES, 2013–2014**				
**Total**	**74.9 (1.8)**	**(71.1–78.4)**	**12.1**	**—**
**Sex**				
Male	78.0 (2.5)	(72.6–82.5)	6.9	reference
Female	71.2 (3.6)	(63.6–77.8)	5.2	0.277
**Age group (yrs)**				
40–64	63.2 (4.5)	(53.9–71.5)	3.5	reference
65–74	78.9 (4.3)	(69.1–86.2)	4.6	0.108
≥65	81.4 (2.7)	(75.4–86.1)	8.8	0.018
≥75	84.8 (3.1)	(77.5–90.0)	4.3	0.004
**Race/Ethnicity**				
White, non-Hispanic	77.9 (1.7)	(74.2–81.1)	9.6	reference
Black, non-Hispanic	80.9 (4.6)	(70.3–88.4)	1.2	0.266
Asian, non-Hispanic	64.3 (8.4)	(46.5–78.8)	0.4	0.116
Hispanic	51.5 (4.4)	(42.8–60.2)	0.7	<0.001
Other	*57.4 (17.4)* ^††^	*(24.9–84.6)* ^††^	0.2	0.242
**Blood pressure control^§§^ among adults aged ≥18 years with hypertension**^¶¶^ **— NHANES, 2015–2016**				
**Total**	**48.5 (2.1)**	**(44.4–52.6)**	**37.9**	**—**
**Sex**				
Male	45.2 (2.7)	(40.0–50.6)	16.9	reference
Female	51.6 (2.7)	(46.4–56.8)	21.1	0.036
**Age group (yrs)**				
18–24	—**	—**	—**	—**
25–44	41.6 (3.1)	(35.6–47.8)	4.4	0.012
18–44	40.0 (3.1)	(34.1–46.1)	4.6	0.004
45–64	53.8 (2.8)	(48.1–59.3)	18.1	reference
65–74	51.5 (3.6)	(44.5–58.4)	8.7	0.307
≥65	45.9 (3.1)	(39.8–52.1)	14.0	0.009
≥75	38.4 (3.3)	(32.1–45.0)	5.2	<0.001
**Race/Ethnicity**				
White, non-Hispanic	50.9 (2.8)	(45.4–56.4)	26.7	reference
Black, non-Hispanic	44.3 (1.6)	(41.2–47.5)	5.1	<0.001
Asian, non-Hispanic	38.2 (4.1)	(30.4–46.6)	1.3	0.012
Hispanic	44.2 (3.0)	(38.3–50.3)	3.9	0.126
Other	46.5 (6.7)	(33.8–59.6)	1.0	0.493
Cholesterol management: statin use*** among eligible adults^†††^ **aged ≥21 years — NHANES, 2013–2014**				
**Total**	**54.5 (1.8)**	**(50.9–58.1)**	**46.9**	**—**
**Sex**				
Male	51.5 (2.1)	(47.3–55.7)	23.8	reference
Female	58.1 (2.5)	(53.0–63.0)	23.1	0.089
**Age group (yrs)^§§^**				
21–24	—**	—**	—**	—**
25–44	37.7 (5.7)	(27.0–49.8)	2.6	0.083
21–44	35.7 (5.4)	(25.6–47.2)	2.7	0.028
45–64	50.3 (2.5)	(45.4–55.3)	21.8	reference
65–74	52.7 (3.0)	(46.5–58.8)	11.8	0.787
≥65	63.5 (2.2)	(59.0–67.8)	22.3	<0.001
≥75	86.2 (3.2)	(78.2–91.6)	10.7	<0.001
**Race/Ethnicity**				
White, non-Hispanic	58.3 (2.1)	(54.0–62.6)	35.8	reference
Black, non-Hispanic	44.3 (4.0)	(36.3–52.5)	4.6	0.013
Asian, non-Hispanic	49.2 (4.0)	(41.2–57.2)	2.0	0.092
Hispanic	33.7 (3.2)	(27.6–40.4)	2.8	<0.001
Other	—**	—**	—**	—**

**Source:** NHANES, National Center for Health Statistics, CDC.

**Abbreviations:** CI = confidence interval; NHANES = National Health and Nutrition Examination Survey; SE = standard error.

* Population counts are calculated using the American Community Survey 2013 or 2015 annual Public Use Microdata Sample files, the latest available file after data collection in the 2013–2014 and 2015–2016 survey cycles, respectively. https://wwwn.cdc.gov/nchs/nhanes/ResponseRates.aspx.

^†^ P-values adjusted for sex, age group, and race/ethnicity using logistic regression.

^§^ Aspirin use was defined by any of the following: an answer of “yes” to the question “Doctors and other health care providers sometimes recommend that you take a low-dose aspirin each day to prevent heart attacks, strokes, or cancer. Have you ever been told to do this?” and an answer of “yes” or “sometimes” to the question “Are you/ now following this advice?”; an answer of “yes” to the question “On your own, are you now taking a low-dose aspirin each day to prevent heart attacks, strokes, or cancer?” Aspirin identified in the Rx medication data files. Participants who reported taking an anticoagulant (as identified in the prescription medication files) but not taking aspirin were excluded.

^¶^ Primary prevention: includes examined adults aged 50–59 years for whom aspirin is recommended by the U.S. Preventive Services Task Force, without a history of cardiovascular (CVD) and with a 10-year atherosclerotic CVD (ASCVD) risk ≥10%. (Bibbins-Domingo K. Aspirin Use for the Primary Prevention of Cardiovascular Disease and Colorectal Cancer: U.S. Preventive Services Task Force Recommendation Statement. Ann Intern Med 2016;164:836–45; U.S. Preventive Services Task Force (USPSTF) Recommendation Summary: https://www.uspreventiveservicestaskforce.org/Page/Document/UpdateSummaryFinal/aspirin-to-prevent-cardiovascular-disease-and-cancer: ASCVD risk score is calculated based on the equations published in Goff DC Jr, Lloyd-Jones DM, et al. 2013 ACC/AHA guideline on the assessment of cardiovascular risk: a report of the American College of Cardiology/American Heart Association Task Force on Practice Guidelines. Circulation. 2014;129:S49–73.) Secondary prevention: includes examined adults aged ≥40 years with a history of cardiovascular disease. A history of CVD is defined as an answer of “yes” to any of the following questions: “Has a doctor or other health professional ever told you that you had angina, also called angina pectoris?”, “Has a doctor or other health professional ever told you that you had coronary heart disease?”, “Has a doctor or other health professional ever told you that you had a heart attack (also called myocardial infarction)?”, “Has a doctor or other health professional ever told you that you had a stroke?”

** Statistically unreliable estimates (relative standard error >40%) are suppressed.

^††^ Estimates are statistically unstable by National Center for Health Statistics standards (relative standard error >30%).

^§§^ Blood pressure (BP) control defined as an average systolic BP <140 mm Hg and an average diastolic BP <90 mm Hg. Calculated among adults with hypertension. Includes non-pregnant examined adults aged ≥18 years with ≥1 complete blood pressure measurement and information to determine BP-lowering medication use.

^¶¶^ Hypertension is defined as an average systolic BP ≥140 mm Hg, or an average diastolic BP ≥90 mm Hg, or self-reported current use of BP-lowering medication. Current use of BP-lowering medication is defined as an answer of “yes” to the questions: “Because of your high blood pressure/hypertension, have you ever been told to take prescribed medicine?” and “Are you currently taking medication to lower your blood pressure?”

*** Statin use is defined using the prescription medication files.

^†††^ Includes non-pregnant fasting adults (≥21 years) for whom a statin is recommended, based on their risk for ASCVD, as defined in: Stone NJ, Robinson J, Lichtenstein AH, et al. 2013 ACC/AHA guideline on the treatment of blood cholesterol to reduce atherosclerotic cardiovascular risk in adults: a report of the American College of Cardiology/American Heart Association Task Force on Practice Guidelines. Circulation 2014;129:S1–45.

During 2015–2016, the prevalence of blood pressure (BP) control was 48.5%, with no significant changes occurring during 2011–2012 (Figure 1). This equates to an estimated 40.2 million persons with uncontrolled hypertension (Supplementary Figure, https://stacks.cdc.gov/view/cdc/58116). The prevalence of BP control was higher among adults aged 45–64 years (53.8%) than among those aged 18–44 years (40.0%) and ≥65 years (45.9%), and among whites (50.9%) than among non-Hispanic blacks (blacks) (44.3%).

The prevalence of cholesterol management through statin use among eligible adults during 2013–2014 was 54.5%, with no significant change occurring during 2011–2012 (Figure 1). Prevalence was higher among persons aged ≥65 years (63.5%) than among those aged 45–64 years (50.3%), and among whites (58.3%) than among Hispanics (33.7%). An estimated 39.1 million adults are not managing their CVD risk through recommended statin use.

Though the prevalence of BP control was higher among adults aged 35–64 years (a Million Hearts priority population) (52.9%) than among those aged ≥65 years (45.9%), still approximately half do not have their condition under control. The prevalence of statin use when indicated among persons aged 35–64 years (48.1%) was lower than that among those aged ≥65 years (63.5%) (Supplementary Table, https://stacks.cdc.gov/view/cdc/58119). 

**FIGURE 2:**
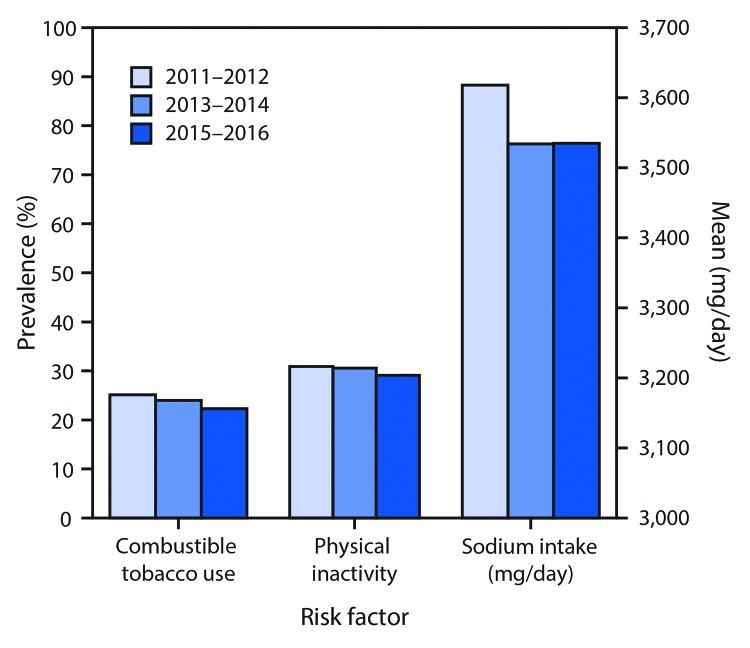
Prevalence of Million Hearts 2022 community risk factors*^,^^†^^,^^§^ for cardiovascular disease among adults^¶^ — United States, 2011–2012, 2013–2014, and 2015–2016 **Source:** National Survey on Drug Use and Health; Substance Abuse and Mental Health Services Administration; National Health and Nutrition Examination Survey; National Center for Health Statistics; CDC; National Health Interview Survey (NHIS). * Combustible tobacco use was defined as current use of combustible tobacco products (cigarettes, cigars, or pipe) among adults (aged ≥18 years) with complete data to determine tobacco use. ^†^ The *2008 Physical Activity Guidelines for Americans* (http://www.health.gov/PAGuidelines/) recommend that all adults should avoid inactivity and that some physical activity is better that none. NHIS questions ask about frequency of participation in light to moderate-intensity and vigorous-intensity leisure-time physical activities for at least 10 minutes. Questions are phrased in terms of current behavior and lack a specific reference period. Physical inactivity is defined as reporting no light to moderate or vigorous leisure-time physical activity for at least 10 minutes. ^§^ Sodium intake (mg/day) was estimated among adults aged ≥18 years with a complete and reliable first day 24-hour dietary recall (collected in-person at the mobile examination center). ^¶^ For combustible tobacco use and physical inactivity, t-test p-values <0.01 comparing 2015–2016 with 2011–2012, adjusted for sex, age group, and race/ethnicity.

**Community Risk Factors.** Despite a significant decline in use of combustible tobacco products, from 25.1% of adults in 2011–2012, to 22.3% during 2015–2016, an estimated 54.1 million adult users of combustible tobacco products could benefit from cessation interventions (Figure 2). During 2015–2016, the prevalence of combustible tobacco use was higher among men (26.7%) than among women (18.1%), decreased with increasing age after age 25–44 years, and varied by race/ethnicity. Prevalence was higher among whites (24.0%) than among Hispanics (16.0%) and non-Hispanic Asians (10.3%); however, persons of “other race/ethnicity,” which includes American Indians and Alaska Natives, reported the highest prevalence (30.8%) of combustible tobacco use (Table 2).

**TABLE 2:** Current prevalence of Million Hearts 2022 community risk factors for cardiovascular disease among adults — United States, 2015–2016

Risk factor/Demographic group	% (SE)	(95% CI)	No. (millions)*	t-test p-value^†^
**Combustible tobacco use among adults aged ≥18 years^§^** **— NSDUH, 2015–2016**				
**Total**	**22.3 (0.2)**	**(21.9–22.7)**	**54.1**	**—**
**Sex**				
Male	26.7 (0.3)	(26.1–27.3)	31.3	reference
Female	18.1 (0.2)	(17.6–18.6)	22.8	<0.001
**Age group^¶^ (yrs)**				
18–24	24.4 (0.4)	(23.7–25.1)	7.5	<0.001
25–44	27.4 (0.3)	(26.8–28.0)	22.7	<0.001
18–44	26.6 (0.2)	(26.1–27.1)	30.2	<0.001
45–64	23.0 (0.4)	(22.2–23.7)	19.1	reference
65–74	13.5 (0.6)	(12.4–14.6)	3.7	<0.001
≥65	10.4 (0.4)	(9.5–11.3)	4.8	<0.001
≥75	5.3 (0.5)	(4.4–6.2)	1.0	<0.001
**Race/Ethnicity**				
White, non-Hispanic	24.0 (0.3)	(23.4–24.6)	37.7	reference
Black, non-Hispanic	24.7 (0.6)	(23.6–25.8)	7.0	0.349
Asian, non-Hispanic	10.3 (0.8)	(8.9–11.9)	1.4	<0.001
Hispanic	16.0 (0.4)	(15.2–16.7)	6.0	<0.001
Other	30.8 (1.0)	(28.9–32.8)	1.9	<0.001
**Physical inactivity among adults aged ≥18 years**^¶^ **— NHIS, 2015–2016**				
**Total**	**29.1 (0.4)**	**(28.3–29.8)**	**70.7**	**—**
**Sex**				
Male	27.3 (0.4)	(26.4–28.2)	31.9	reference
Female	30.7 (0.5)	(29.9–31.6)	38.7	<0.001
**Age group^¶^ (yrs)**				
18–24	22.5 (0.9)	(20.9–24.3)	6.9	<0.001
25–44	23.6 (0.5)	(22.6–24.5)	19.5	<0.001
18–44	23.3 (0.5)	(22.4–24.2)	26.4	<0.001
45–64	30.1 (0.5)	(29.0–31.2)	25.0	reference
65–74	34.2 (0.7)	(32.9–35.6)	9.3	<0.001
≥65	41.2 (0.6)	(40.0–42.3)	19.1	<0.001
≥75	51.2 (0.8)	(49.5–52.8)	9.8	<0.001
**Race/Ethnicity**				
White, non-Hispanic	26.4 (0.4)	(25.6–27.3)	41.5	reference
Black, non-Hispanic	36.9 (0.8)	(35.3–38.6)	10.5	<0.001
Asian, non-Hispanic	24.6 (1.4)	(22.0–27.5)	3.3	0.916
Hispanic	36.1 (0.9)	(34.3–38.0)	13.6	<0.001
Other	24.5 (1.3)	(22.1–27.0)	1.5	0.828
**Risk factor/Demographic group**	**Mean (SE)**	**(95% CI)**	No. (millions)*	**p-value^†^**
Average dietary sodium intake (mg/day) among adults aged ≥18 years** **— NHANES, 2015–2016**				
**Total**	**3,535 (41)**	**(3,452–3,618)**	**N/A**	**—**
**Sex**				
Male	4,095 (65)	(3,964–4,226)	N/A	reference
Female	3,013 (38)	(2,936–3,089)	N/A	<0.001
**Age group^¶^ (yrs)**				
18–24	3,733 (109)	(3,515–3,951)	N/A	0.1205
25–44	3,834 (75)	(3,683–3,985)	N/A	<0.001
18–44	3,809 (68)	(3,673–3,946)	N/A	<0.001
45–64	3,524 (50)	(3,424–3,625)	N/A	reference
65–74	3,092 (96)	(2,899–3,284)	N/A	<0.001
≥65	2,947 (66)	(2,815–3,078)	N/A	<0.001
≥75	2,733 (92)	(2,549–2,918)	N/A	<0.001
**Race/Ethnicity**				
White, non-Hispanic	3,515 (54)	(3,406–3,624)	N/A	reference
Black, non-Hispanic	3,364 (60)	(3,243–3,484)	N/A	0.0047
Asian, non-Hispanic	3,831 (114)	(3,601–4,062)	N/A	0.0632
Hispanic	3,582 (65)	(3,450–3,713)	N/A	0.3540
Other	3,726 (283)	(3,156–4,296)	N/A	0.6184

**Sources:** NSDUH; Substance Abuse and Mental Health Services Administration; NHANES; National Center for Health Statistics; CDC National Health Interview Survey (NHIS); NCHS; CDC.

**Abbreviations:** CI = confidence interval; N/A = not applicable; NHANES = National Health and Nutrition Examination Survey; NSDUH = National Survey on Drug Use and Health; SE = standard error.

* Population counts are calculated using the American Community Survey 2013 or 2015 annual Public Use Microdata Sample files, the latest available file after data collection in the 2013–2014 and 2015–2016 survey cycles, respectively. https://wwwn.cdc.gov/nchs/nhanes/ResponseRates.aspx.

^†^ P-values adjusted for sex, age group, and race/ethnicity using logistic regression.

^§^ Includes current use of combustible tobacco products (cigarettes, cigars, or pipes) among adults (≥18 years). Current cigarette smoking defined as an answer of “yes” to the question “Have you smoked at least 100 cigarettes in your entire life?” and an answer of “Within the past 30 days” to the question “How long has it been since you last smoked part or all of a cigarette?” Current cigar smoking defined as an answer of “Within the past 30 days” to the question “How long has it been since you last smoked part or all of any type of cigar?” Current pipe smoking defined as an answer of “yes” to the question “During the past 30 days, have you smoked tobacco in a pipe, even once?”

^¶^ The *2008 Physical Activity Guidelines for Americans* (https://www.health.gov/PAGuidelines/) recommend that all adults should avoid inactivity and that some physical activity is better that none. NHIS questions ask about frequency of participation in light to moderate-intensity and vigorous-intensity leisure-time physical activities for at least 10 minutes. Questions are phrased in terms of current behavior and lack a specific reference period. Physical inactivity is defined as reporting no light to moderate or vigorous leisure-time physical activity for at least 10 minutes.

** Includes adults (aged ≥18 years) with a complete and reliable 1st day 24-hour dietary recall (collected in-person at the mobile examination center). Sodium values are not adjusted for salt added during food preparation or at the table.

During 2015–2016, the mean daily sodium intake among adults was 3,535 mg/day, with no significant change occurring from 2011–2012 (Figure 2). Sodium intake was higher among men (4,095 mg/day) than among women (3,013 mg/day) and decreased with increasing age, from 3,809 mg/day for persons aged 18–44 years to 3,524 mg/day for those aged 45–64 years, and 2,947 mg/day among adults aged ≥65 years.

During 2015–2016, the prevalence of physical inactivity was 29.1%, a small but statistically significant decrease from 30.9% during 2011–2012 (Figure 2). This represents an estimated 70.7 million adults who currently partake in no leisure time physical activity. The prevalence of physical inactivity was higher among women (30.7%) than among men (27.3%), increased with increasing age, and was higher among blacks (36.9%) and Hispanics (36.1%) than among whites (26.4%).

Among the Million Hearts priority population of adults aged 35–64 years, the prevalence of combustible tobacco product use and average daily sodium intake were higher than those among adults aged ≥65 years, while the prevalence of physical inactivity was lower (Supplementary Table, https://stacks.cdc.gov/view/cdc/58119).

## Conclusion and Comment

To reach the Million Hearts 2022 goal of preventing one million acute cardiovascular events over 5 years, substantial progress is needed in reducing CVD-related risk factors. To achieve needed progress, Million Hearts 2022 has set clinical targets of 80% performance on the “ABCS” of CVD prevention: aspirin when appropriate, blood pressure control, cholesterol management, and smoking cessation. At the community level, a 20% reduction in the prevalence of combustible tobacco product use and of physical inactivity and a 20% reduction in mean daily sodium intake are targeted. These indicators, along with cardiac rehabilitation participation, are the focus of Million Hearts 2022; progress in reaching indicator targets has been shown to have a substantial effect on preventing acute cardiovascular events (*11**,**12*).

The data in this report serve as a baseline for Million Hearts 2022. These findings suggest that in addition to universal strategies aimed at the entire population with and at risk for CVD, there is a need to focus action on high-burden, high-risk subsets of the population. For example, opportunities for risk factor prevention and management among younger adults are of particular importance given the increase in heart disease mortality observed from 2010 to 2015 among adults aged 35–64 years in approximately half of U.S. counties (*3*). Compared with adults aged ≥65 years, younger adults were less likely to be using aspirin or taking a statin when indicated and were more likely to use combustible tobacco and have an elevated daily sodium intake. Furthermore, only approximately half of adults aged 35–64 years with hypertension have their BP under control. If the population deficits for each risk factor in this analysis (e.g., 9.0 million persons who are not taking aspirin as recommend) are summed, they represent approximately 213 million opportunities for better risk factor prevention and management, many of which might be present in the same person. More than half of these opportunities are among adults aged 35–64 years.

Additional demographic disparities in risk factor prevalence present opportunities to develop and implement culturally and linguistically tailored and effective interventions. For example, compared with whites, Hispanics were less likely to use aspirin for secondary prevention or take a statin when indicated, blacks were less likely to have their blood pressure under control, and persons of “other” racial/ethnic groups, including American Indians and Alaska Natives, were more likely to use combustible tobacco products. Other studies confirm the existence of these disparities (*13**–**15*).

Included in the Million Hearts 2022-recommended clinical strategies are self-measured blood pressure monitoring with clinical support,**** standardized treatment protocols,^††††^ reduced out-of-pocket costs^§§§§^ and adherence approaches^¶¶¶¶^ for medications, clinician-driven tobacco assessment and treatment,***** increasing awareness of the effect of particle pollution (including tobacco smoke, automobile or diesel exhaust, and wood smoke)^†††††^ on persons with known heart disease, and using clinical data to identify persons with undiagnosed conditions.^§§§§§^ Community-based strategies include comprehensive smoke-free policies,^¶¶¶¶¶^ evidence-based tobacco cessation campaigns,****** sodium reduction strategies,^††††††^ built environment approaches^§§§§§§^ to increase physical activity, increased access to places for physical activity,^¶¶¶¶¶¶^ and peer support programs.******* Public and private partners, such as the Agency for Healthcare Research and Quality’s EvidenceNOW initiative,^†††††††^ state and local departments of health, the National Association of Community Health Centers, and Target:BP^§§§§§§§^ from the American Heart Association and the American Medical Association, are actively implementing these strategies.

The findings in this report are subject to at least five limitations. First, some data used in this analysis are self-reported and subject to recall and social desirability biases. Second, when the data assessing aspirin use for primary CVD prevention were collected, multiple clinical guidelines existed; definitions from the current U.S. Preventive Services Task Force recommendation, published in 2016, were retrospectively applied for this analysis. Third, the American College of Cardiology and American Heart Association’s (ACC/AHA) cholesterol management guideline, released in November 2013 with a focus on high-intensity statin use (treatment that lowers low-density lipoprotein cholesterol by approximately ≥50%) among eligible persons at high risk for cardiovascular events, was retrospectively applied to the 2011–2012 data. NHANES data allow for reporting only on general statin use and not statin intensity, which might result in overestimating the prevalence of the statin-eligible population meeting recommendations. Fourth, Million Hearts focuses on the “ABCS,” which include smoking cessation through assessment and treatment in a clinical setting. However, population-level data for this indicator do not exist so only combustible tobacco use prevalence can be monitored, but not clinical actions that support cessation. Finally, as with many national data collection efforts, there is a 2–3 year data lag for some indicators. As a result, incongruous data cycles are reported in this analysis.

Heart disease and stroke are leading causes of death in the United States; their risk factors are prevalent in the general population and are particularly high among certain subgroups. Evidence-based strategies for preventing acute cardiovascular events exist, with 213 million opportunities for better risk factor prevention and management. It will require a concerted national implementation effort to prevent one million acute cardiovascular events by 2022.

**Summary**

**What is already known about this topic?**

The decline in cardiovascular disease (CVD) mortality rate has begun to plateau in the general population and has increased among some subpopulations; the prevalence of CVD risk factors remains high. Million Hearts 2022 was launched to focus the nation on high-impact, evidence-based strategies to prevent one million acute cardiovascular events over five years.

**What is added by this report?**

During 2015–2016, adult sodium intake averaged 3,535 mg/day and the prevalences of blood pressure control, combustible tobacco use, and physical inactivity were 48.5%, 22.3% and 29.1%, respectively. Compared with 2011–2012, significant improvements were observed in combustible tobacco use and physical inactivity, but the prevalence of aspirin use to prevent CVD declined.

**What are the implications for public health practice?**

A concerted effort to implement evidence-based strategies is needed to achieve the Million Hearts 2022 goal.

## Footnotes

* Although participation in cardiac rehabilitation is an evidence-based strategy for preventing secondary CVD-related events, it is not considered a key CVD risk factor. Therefore the participation data are not included in this report.

† During 2011–2016, unweighted examination response rates ranged from 58.7% to 69.5%. https://www.cdc.gov/nchs/nhanes.htm.

§ During 2011–2016, weighted interview response rates ranged from 71.2% to 74.4%. https://datafiles.samhsa.gov/study/national-survey-drug-use-and-health-nsduh-2011-nid13563; https://datafiles.samhsa.gov/study/national-survey-drug-use-and-health-nsduh-2012-nid13601; https://datafiles.samhsa.gov/study/national-survey-drug-use-and-health-nsduh-2013-nid13555; https://datafiles.samhsa.gov/study/national-survey-drug-use-and-health-nsduh-2014-nid13618; https://datafiles.samhsa.gov/study/national-survey-drug-use-and-health-nsduh-2015-nid16893; https://datafiles.samhsa.gov/study/national-survey-drug-use-and-health-nsduh-2016-nid17184.

¶ During 2011–2016, the final response rate for the sample adult component ranged from 79.7%–81.7%. https://www.cdc.gov/nchs/nhis.htm.

** The U.S. Preventive Services Task Force recommends initiating low-dose aspirin use for the primary prevention of CVD in adults aged 50–59 years who have no history of CVD, a ≥10% 10-year atherosclerotic CVD (ASCVD) risk, and are not at increased risk for bleeding (https://www.uspreventiveservicestaskforce.org/Page/Document/RecommendationStatementFinal/aspirin-to-prevent-cardiovascular-disease-and-cancer). Aspirin use in NHANES was defined by self-report or aspirin identified in the prescription medication data files. Participants who were taking an anticoagulant but not taking aspirin/antiplatelets were excluded as being at increased risk for bleeding. Participants who reported that they stopped taking aspirin because of side effects were excluded. During 2011–2014, among 2,776 adults aged 50–59 years examined in NHANES with complete data to determine aspirin eligibility, 338 met the criteria for aspirin use for primary prevention.

†† Aspirin use for secondary event prevention is recommended for adults aged ≥40 years with a history of CVD, defined as self-reported angina, coronary heart disease, heart attack, or stroke. Aspirin use in NHANES was defined by self-report or aspirin identified in the prescription medication data files. During 2011–2014, among 11,184 adults aged ≥40 years examined in NHANES with complete data to determine aspirin eligibility, 913 met the criteria for aspirin use for secondary prevention.

§§ “Aspirin when appropriate” is defined as primary or secondary prevention use among eligible adults. During 2011–2014, 1,251 adults ≥40 years were included in the aspirin when appropriate analyses.

¶¶ The 2013 ACC/AHA guideline recommends statin treatment for persons 1) with clinical atherosclerotic CVD (ASCVD); 2) with low-density lipoprotein cholesterol (LDL-C) ≥190 mg/dL; 3) aged 40–75 years with diabetes, LDL-C 70–189 mg/dL, and without clinical ASCVD; or 4) aged 40–75 years without clinical ASCVD or diabetes with LDL-C 70–189 mg/dL, and estimated 10-year ASCVD risk ≥7.5% (https://www.ahajournals.org/doi/abs/10.1161/01.cir.0000437738.63853.7a; https://www.ahajournals.org/doi/abs/10.1161/01.cir.0000437741.48606.98). Statin use was identified using the NHANES prescription medication data files. During 2011–2014, among 4,358 non-pregnant adults aged ≥21 years in the morning fasting subsample in NHANES with complete data to determine statin eligibility, 1,823 met the criteria for statin use.

*** Dietary sodium intake is estimated from the NHANES Day 1 dietary recall interviews (https://www.ars.usda.gov/ba/bhnrc/fsrg). During 2011–2016, 15,698 adults aged ≥18 years had a complete and reliable Day 1 dietary recall and were included in the sodium analyses.

††† Defined among adults with hypertension as systolic BP of <140 mm Hg and diastolic BP of <90 mm Hg, based on the average of up to three measurements. ACC/AHA released a new hypertension management guideline in November 2017 that uses 130/80 mm Hg to define blood pressure control (https://professional.heart.org/professional/ScienceNews/UCM_496965_2017-Hypertension-Clinical-Guidelines.jsp). Here, 140/90 mm Hg is used to define control because that was the standard, as recommended for the general population by the Seventh Joint National Committee on Prevention, Detection, Evaluation, and Treatment of High Blood Pressure, when these data were collected (Chobanian A V, Bakris G,Black H, et al. The Seventh Report of the Joint National Committee on Prevention, Detection, Evaluation, and Treatment of High Blood Pressure: the JNC 7 report. JAMA 2003;289:2560-2572). Among the participants, approximately 95% had two or three blood pressure measurements during a single physical examination at the mobile examination center. For the remainder with only one blood pressure measurement, that single measurement was used in place of an average. During 2011–2016, of the 16,457 nonpregnant adults aged ≥18 years examined in NHANES with complete blood pressure and medication data, 5,765 were defined as hypertensive and included in the blood pressure analyses.

§§§ Combustible tobacco use includes the use of cigarettes, cigars, or pipes. The percentage of adults aged ≥18 years who reported smoking cigarettes on at least 1 day during the preceding 30 days and ≥100 cigarettes in their lifetime, or who reported smoking cigars or a pipe on at least 1 day during the preceding 30 days. During 2011–2016, of the 242,283 persons aged ≥18 years included in the NSDUH population, 241,799 were included in the current combustible tobacco use analyses.

¶¶¶ The *2008 Physical Activity Guidelines for Americans* (https://www.health.gov/PAGuidelines/) recommend that all adults should avoid inactivity and that some physical activity is better that none. NHIS questions ask about frequency of participation in light to moderate-intensity and vigorous-intensity leisure-time physical activities for at least 10 minutes. Questions are phrased in terms of current behavior and lack a specific reference period. Physical inactivity is defined as reporting no light to moderate or vigorous leisure-time physical activity for at least 10 minutes. During 2011–2016, of the 205,493 adults aged ≥18 years included in the NHIS population, 202,941 were included in the physical inactivity analyses.

**** https://millionhearts.hhs.gov/tools-protocols/smbp.html.

†††† https://millionhearts.hhs.gov/tools-protocols/protocols.html.

§§§§ https://www.thecommunityguide.org/findings/cardiovascular-disease-reducing-out-pocket-costs-cardiovascular-disease-preventive-services; https://www.thecommunityguide.org/findings/tobacco-use-and-secondhand-smoke-exposure-reducing-out-pocket-costs-evidence-based-cessation.

¶¶¶¶ https://millionhearts.hhs.gov/tools-protocols/medication-adherence.html.

***** https://millionhearts.hhs.gov/files/Tobacco-Cessation-Action-Guide.pdf.

††††† https://millionhearts.hhs.gov/tools-protocols/tools/particle-pollution.html.

§§§§§ https://millionhearts.hhs.gov/tools-protocols/hiding-plain-sight/index.html.

¶¶¶¶¶ https://www.thecommunityguide.org/findings/tobacco-use-and-secondhand-smoke-exposure-smoke-free-policies.

****** https://www.thecommunityguide.org/findings/tobacco-use-and-secondhand-smoke-exposure-mass-reach-health-communication-interventions; https://www.cdc.gov/tobacco/campaign/tips/index.html

†††††† https://www.cdc.gov/vitalsigns/sodium.

§§§§§§ https://www.thecommunityguide.org/findings/physical-activity-built-environment-approaches.

¶¶¶¶¶¶ https://www.thecommunityguide.org/findings/physical-activity-creating-or-improving-places-physical-activity.

******* https://www.thecommunityguide.org/findings/physical-activity-social-support-interventions-community-settings.

††††††† https://www.ahrq.gov/evidencenow/index.html.

§§§§§§§ https://targetbp.org/.
